# Distributed synaptic plasticity controls spike-timing: predictions from a cerebellar computational model

**DOI:** 10.1186/1471-2202-14-S1-P81

**Published:** 2013-07-08

**Authors:** Jesús A Garrido, Eduardo Ros, Egidio D'Angelo

**Affiliations:** 1 Dept. of Brain and Behavioral Sciences, University of Pavia, Consorzio Interuniversitario per le Scienze Fisiche della Materia (CNISM), Pavia, 27100, Italy; 2 Dept. of Computer Architecture and Technology, University of Granada, Granada, Spain; 3 Brain Connectivity Center (BCC), IRCCS C.Mondino, Via Mondino 2, I-27100 Pavia, Italy

## 

The cerebellum has long been proposed to operate as a "timing machine" and a "learning machine", but the intrinsic nature of these operations has not been resolved yet. Interestingly, the cerebellum controls motor behavior with millisecond precision (e.g. in a throwing task), so it is expected that its computations are performed on a comparable time-scale. There are indications that the granular layer is capable of exerting a close control on spike timing. The granule cells generate brief spike bursts in the axon initial segment, which are almost instantaneously (<0.3 ms) transmitted to the dendrites and to synapses on the axonal ascending branch [1]. Moreover, the mossy fiber-granule cell EPSCs have very fast kinetics (rise time <1 ms) [2] and can therefore excite the granule cells with high temporal precision [3]. Finally, granule cells are endowed with specific ionic mechanisms capable of controlling the delay and persistence of spike emission [4]. Interestingly, LTP and LTD have been shown to regulate both first-spike delay and spike frequency through different mechanisms [5]. The outstanding timing capabilities of this system have been summarized into the "time-window" hypothesis, which considers how these mechanisms compete with feed-forward synaptic inhibition mediated by Golgi cells in order to control spike emission from the granule cells during a period of a few milliseconds after mossy fiber burst discharge [6].

In order to investigate how plasticity distributed over different granular layer synapses affects spike-timing, we have simulated the impact of multiple synaptic weight changes in the granular layer (EDLUT spiking simulator). In response to spike bursts, the granular-layer network model generated a limited number of spikes with sub-millisecond precision. Synaptic weights at multiple connections played a crucial role to regulate spike number and positioning. In particular, mossy fiber to granule cell synapse regulated the delay of the response, mossy fiber and parallel fiber to Golgi cell synapses regulated the duration of the permissive time-window, while Golgi cell to granule cell synapse regulated the duration of the inhibitory time-window. Moreover, inhibitory connections to Golgi cells (either from other Golgi or from stellate cells) controlled the number of spikes (from 0 to 3) in response to burst stimulation. These results predict that distributed synaptic plasticity enhances the computational capabilities of the granular layer, which can then operate as a complex adaptable temporal filter on the millisecond time-scale.

## References

[B1] Diwakar S Lombardo P Solinas S Naldi G D'Angelo E Local field potential modeling predicts dense activation in cerebellar granule cells clusters under LTP and LTD control PLoS One 2011 6 7 e21928 10.1371/journal.pone.0021928 21818278 PMC3139583

[B2] Silver RA Traynelis SF Cull-Candy SG Rapid-time-course miniature and evoked excitatory currents at cerebellar synapses in situ Nature 1992 335 163 166 1370344 10.1038/355163a0

[B3] Cathala L Brickley S Cull-Candy S Farrant M Maturation of EPSCs and intrinsic membrane properties enhances precision at a cerebellar synapse The Journal of neuroscience 2003 23 14 6074 6085 12853426 10.1523/JNEUROSCI.23-14-06074.2003 PMC6740347

[B4] D'Angelo E Nieus T Maffei A Armano S Rossi P Taglietti V Fontana A Naldi G Theta-frequency bursting and resonance in cerebellar granule cells: experimental evidence and modeling of a slow K+-dependent mechanism The Journal of Neuroscience 2001 21 3 759 11157062 10.1523/JNEUROSCI.21-03-00759.2001 PMC6762330

[B5] Roggeri L Rivieccio B Rossi P D'Angelo E Tactile stimulation evokes long-term synaptic plasticity in the granular layer of cerebellum J Neurosci 2008 28 25 6354 6359 10.1523/JNEUROSCI.5709-07.2008 18562605 PMC6670905

[B6] D'Angelo E De Zeeuw CI Timing and plasticity in the cerebellum: focus on the granular layer Trends in neurosciences 2009 32 1 30 40 10.1016/j.tins.2008.09.007 18977038

